# Predicting postoperative complications of head and neck squamous cell carcinoma in elderly patients using random forest algorithm model

**DOI:** 10.1186/s12911-015-0165-3

**Published:** 2015-06-09

**Authors:** YiMing Chen, Wei Cao, XianChao Gao, HuiShan Ong, Tong Ji

**Affiliations:** Department of Oral Maxillofacial-Head Neck Oncology, Ninth People’s Hospital, School of Medicine, Shanghai Jiao Tong University, 639 Zhizaoju Road, Shanghai, 200011 China

**Keywords:** Head and neck squamous cell carcinoma (HNSCC), Postoperative complications, Predictive model, Data mining (DM), Elderly patients

## Abstract

**Background:**

Head and Neck Squamous Cell Carcinoma (HNSCC) has a high incidence in elderly patients. The postoperative complications present great challenges within treatment and they're hard for early warning.

**Methods:**

Data from 525 patients diagnosed with HNSCC including a training set (*n* = 513) and an external testing set (*n* = 12) in our institution between 2006 and 2011 was collected. Variables involved are general demographic characteristics, complications, disease and treatment given. Five data mining algorithms were firstly exploited to construct predictive models in the training set. Subsequently, cross-validation was used to compare the different performance of these models and the best data mining algorithm model was then selected to perform the prediction in an external testing set.

**Results:**

Data from 513 patients (age > 60 y) with HNSCC in a training set was included while 44 variables were selected (*P* < 0.05). Five predictive models were constructed; the model with 44 variables based on the Random Forest algorithm demonstrated the best accuracy (89.084 %) and the best AUC value (0.949). In an external testing set, the accuracy (83.333 %) and the AUC value (0.781) were obtained by using the random forest algorithm model.

**Conclusions:**

Data mining should be a promising approach used for elderly patients with HNSCC to predict the probability of postoperative complications. Our results highlighted the potential of computational prediction of postoperative complications in elderly patients with HNSCC by using the random forest algorithm model.

## Background

Head and neck carcinoma (HNC) represents the sixth most common cancer worldwide [1], and squamous cell carcinoma (SCC) is the most common type of HNC. Surgery is regarded as one of the most effective treatments for HNSCC [1]. However, the postoperative complications present serious challenges for every surgeon in the management of patients with HNSCC, especially for elderly patients.

Many aspects contribute to the high risk of postoperative complications for elderly patients with HNSCC, but in general, it has been reported that patients over the age of 70 are more likely to experience postoperative complications [2] than younger patients. Poor physical health and poor living habits also confer a disadvantage, and Warner et al. contended that nonsmokers who undergo Coronary Artery Bypass Grafting (CABG) have a 66 % reduction in the risk for postoperative complications in the lung compared with smokers [3]. Cardiovascular complications are also significant causes of postoperative morbidity and mortality in patients undergoing surgery [4]. Co-morbidities are considered prognostic factors in studies of the benefit quality and the effectiveness of care where postoperative complications are the focus of discussion [5].

The following characteristics of treatment, especially the surgeries that are performed, are also influential factors for patients: the duration, the scale and the level of surgical operations. Fleisher et al. have found that patients who are ASA III and IV according to the American Society of Anesthesiologist, have a death rate of 13 and 26 % respectively [6].

The most widely used systems for predicting potential postoperative complications are the Acute Physiology and Chronic Health Evaluation II system (APACHE II) [7], the Physiological and Operative Severity Score for Enumeration of Mortality and Morbidity system (POSSUM) [8], and the American Society of Anesthesiologists (ASA) Physical Status Classification [9]. There are shortcomings in the aforementioned systems, such as the absence of choice for a surgical procedure, length of the procedure, a lack of a description of the surgical procedure, a lack of attention to the nutrition condition of the patients as well as the physiological state of the cardiovascular system.

Due to the development of computational technology, Data Mining (DM) now plays an important role in medical data analysis and modeling [10]. In the process of data mining, three steps should be performed to recognize the rules and patterns among these enormous data sets: 1) Data preparation—collect the data under established inclusion criteria; 2) Data modeling and mining—find the logistic relationships among the data with the use of data mining algorithms; and 3) Results analysis—verify the data mining results to see whether they meet the needs of the study and whether the results are accurate and reliable [11]. Thus far, data mining has been widely used in the medical assessment of various cancer types. For instance, Roberta et al. found 40 genes that are up-regulated in HNSCC using ORESTES data mining analysis [12]. Zainab Abu Bakar et al. constructed a framework for the diagnosis of the tumor stage of primary oral cancer by using data mining of Bayesian reasoning [13].

However, there is lack of computational models that can be used to predict the postoperative complications that may occur in elderly patients with HNSCC. More specifically, there are rarely studies for Data Mining algorithm modelling of postoperative complications with HNSCC. In order to bridge the gap efficiently, three specific aims of the study have been proposed: 1) to design and implement a retrospective cohort study and enroll a sample of elderly patients with HNSCC; 2) to adapt data mining protocols to the study; and 3) to construct predictive models for the postoperative complications based on data mining, to check the reliability of those models and to select the optimal model for predicting postoperative complications in elderly HNSCC patients.

## Methods

### Patients’ data collection

This study had gained ethical approval from the department of Oral and Maxillofacial-Head Neck Oncology at the Affiliated Ninth People’s Hospital School of Medicine, Shanghai Jiao Tong University; The patients’ data were analyzed and collected from the medical records that were documented in the department above; Total 525 patients diagnosed with HNSCC including a training set (*n* = 513) and an external test set (*n* = 12) in our institution between 2006 and 2011 was recruited. The inclusion criteria are: 1) patients older than 60 years; 2) have a pathologically confirmed diagnosis of HNSCC; 3) have no history of other types of tumors; 4) undergo surgery with general anesthesia; and 5) undergo surgery with a curative intent that involved regions within the head and neck. Patients who underwent 1) surgery for benign tumors; 2) surgery for minor procedures with local anesthesia, such as biopsies; and 3) surgery with a duration that was shorter than 30 min were excluded from the study.

### Variable selection

For variable selection, the variables within two operative severity scoring systems were adopted: Acute Physiology and Chronic Health Evaluation II (APACHE II) 7, and Physiological and Operative Severity Score for the enumeration of mortality and morbidity (POSSUM) [8]. Moreover, the variables of the types of surgery and the general condition were further selected through clinical knowledge and experience, and the variables related to poor living habits were evaluated by an SPSS toolkit (*P* < 0.05).

With respect to surgical procedures, as well as the standards of POSSUM score system, the types of surgery were classified into three groups: 1) Minor Operation (MO), primary closure of wounds without neck dissection; 2) Intermediate Operation (IO), primary closure of wounds with unilateral neck dissection; 3) Major operation (JO), primary closure of wounds with bilateral neck dissection or simultaneous flap repair. 4) Superior Major Operation (SMO), primary closure of wounds with bilateral neck dissection and simultaneous flap repair (using microsurgical vascular anastomosis).

Although all the variables are selected from the two operative severity scoring systems which are well accepted worldwide, our study also calculated the information gain (IG) for each variable to determine its effectiveness in a classification system. As listed in Table 1, most scores are very consistent with the clinical experience. Yet, few variables have low IG, which doesn't mean these variables are not important for classification results. For example, IG for rectal temperature is 0.01, but the variable is well used in the general surgery while our study focused on the region of head and neck. From our clinical experience, it still has potential relationship with postoperative complications. The effectiveness of such variables are not as much as the others, but none of them should be deleted.

**Table 1:** The information gain (IG) of 44 variables

Variable	IG	Variable	IG	Variable	IG	Variable	IG
Age	4.427	GCS	0.000	Respiratory function	2.971	The chronic diseases	17.430
Cardiac function	2.908	Operation type	1.863	Levels of sodium	1.373	Smoking	1.813
Respiratory function	2.971	Number of procedures	2.009	Potassium	4.144	Alcoholism	1.170
ECG	3.388	Operative blood loss	13.551	Creatinine	0.928	Preoperative radiotherapy	1.053
Systolic BP	3.72	Peritoneal contamination	1.661	Hematocrit	0.980	Preoperative chemotherapy	1.465
Pulse rate	2.981	Malignancy	2.477	Preoperative WBC	4.002	Preoperative surgery	2.099
Hemoglobin levels	4.511	Confidential Enquiry into Perioperative Deaths	0.015	Operation time	17.060	Primary/recurrence	1.745
WBC count	4.002	Rectal temperature	0.010	Diameter of the tumor	10.935	Surgery classification	16.009
Levels of blood urea nitrogen	0.927	Mean arterial pressure:	2.103	Amount of blood loss	13.551	Clinical grading	8.2800
Sodium:	3.291	Blood pH	2.372	Hematocrit of POD1	1.983	Pathological grading:	4.998
Potassium	5.370	Heart rate	2.981	Glucose of POD1	9.560	Preoperative blood urea (g/L)	0.913

### Postoperative complications selection

The following postoperative complications were selected from the Adult Comorbidity Evaluation-27 (ACE-27) [14]. The other postoperative complications were selected through clinical experiences, especially for the flap reconstruction and tracheotomy as well as for the need of behavior management: infection (wound infection and free flap infection), edema, wound dehiscence, hematoma (with or without re-exploration), free flap necrosis (partial and total), respiratory disorder (pneumonia and pulmonary infarct), salivary fistula, gastrointestinal disorder (abdominal discomfort and hematemesis), deep vein thromphlebitis, angina, and delirium.

### Algorithms for data mining

Five data mining algorithms were exploited in this study: Bayes Net, Naïve Bayes (NB) Net, Support Vector Machine (SVM), Random Forest (RF), and Rotation Forest (ROF) [15-17]. The Naïve Bayes (NB) Net, which is based on Bayes’ theorem, is known as the simplest probabilistic classifier with an assumption that there is no coupling relation between variable spaces. Support Vector Machine (SVM) is a machine learning approach that is based on the structural risk minimization principle of statistics learning theory that is applied in many complicated modeling problems; the decision tree algorithm demonstrates a tree-like graph or model of decisions and their possible consequences, where the sequence of a node is determined by the information entropy of the corresponding node because these variables are mapped to tree nodes in advance. The Random Forest (RF) and Rotation Forest (ROF) algorithms are two improved versions of the decision tree algorithm. In our study, all of the above data mining algorithms were used to construct the predictive models through software Orange 2.7.2 (Version 2.7.2, freely available at http://www.ailab.si/orange/).

### Validation of predictive models and variables

Before a predictive model is constructed, its validity and reliability must be evaluated. The N-fold-cross-validation is a effectively optimal parameter selection tool for model construction and popular measurement tool for model evaluation. Firstly, each sub-dataset in the entire training dataset is singled out, in turn, as the testing sample followed by the remaining N-1 sub-datasets that constitute the new training dataset; this workflow continues until all of the sub-datasets in the complete training dataset are traversed. In this study, N is set to different values such as 3, 5, 10 or more extremely, *N* = 513 (leave-one-out cross-validation). Subsequently, the complete training dataset have been divided into N parts and then optimal model parameters have been selected from grid search based on model performance comparison. It is found that when *N* > =5, the optimal model parameters from grid search approximately keep consistent, which means that model optimization iterative process has stable convergence. Therefore, N was set to five and the Receiver Operating Characteristic (ROC) and the area under the ROC curve (AUC) were adapted to check the performance of all five predictive models. In the study, the collected data were subjected to analysis using SPSS (SPSS17.0) and the level of statistical significance was set at 5 % (*P* < 0.05). Furthermore, univariate analysis was performed firstly with the f-test and t-test for all selected variables; only the significant variables according to the analysis were included.

## Results

### Patient’s data from two independent sets were collected

In training set, Of the 513 patients, 52.0 % were men, and 48.0 % were women. The mean age of the sample was 71.0 years, while the age range was 61–101 years. Clinicopathological characteristic of the patients were listed in Table 2.

**Table 2:** Clinicopathological characteristic of the patients in training set

Characteristic	No.(%) of patients
Gender	Total: 513
Male	266(52.0 %)
Female	247(48.0 %)
Primary/recurrence	Total: 513
Primary	342(66.6 %)
Recurrence	171(33.3 %)
Co-morbidities	Total: 173
High blood pressure	45(26.6 %)
Myocardial infarction	34(20.0 %)
COPD	11(6.4 %)
Bronchitis	13(7.5 %)
Diabetes	31(17.9 %)
Hypothyroidism	10(5.8 %)
Hyperthyroidism	3(1.7 %)
Agitation	5(2.9 %)
Delirium	3(1.7 %)
Metabolism	6(3.5 %)
Acid reflux	6(3.5 %)
Renal failure	6(3.5 %)
Pathological grading	Total: 513
I	148(28.8 %)
II	262(51.0 %)
III	113(22.0 %)
Unidentified	47(9.0 %)
Clinical grading	Total: 513
T1	148(%)
T2	262(%)
T3	47(%)
T4	56(%)
Region	Total: 513
Upper lip	5(1.0 %)
Lower lip	15(2.9 %)
Maxillofacial	17(3.3 %)
Floor of mouth	33(6.4 %)
Tongue	122(23.4 %)
Oral-pharyngeal	12(2.5 %)
Hypopharynx	12(2.5 %)
Neck	49(9.6 %)
Maxilla	52(10.1 %)
Mandible	123(24.0 %)
Buccal mucosa	65(12.7 %)
Parotid gland	6(1.2 %)
Surgical classification	Total: 513
IO	149(30.9 %)
JO	261(50.1 %)
SMO	103(20.0 %)
Postoperative complications	Total: 292
Wound infection	35(12.0 %)
Free flap infection	23(4.5 %)
Edema	46(15.8 %)
Wound dehiscence	40(13.7 %)
Hematoma with re-exploration	15(5.5 %)
Hematoma without re-exploration	10(3.4 %)
Partial flap necrosis	17(5.8 %)
Total flap necrosis	4(1.4 %)
Pneumonia	19(4.8 %)
Pulmonary embolus	1(0.3 %)
Salivary fistula	16(5.5 %)
Abdominal discomfort	5(1.7 %)
Haematemesis	4(1.4 %)
Central nerve system co-morbidity	8(2.7 %)
Deep venous thrombosis (DVT)	7(2.4 %)
Angina	6(2.1 %)
Delirium	8(2.7 %)

A total of 173 patients were found to have co-morbidities: 45.7 % (45: high blood pressure, 34: myocardial infarction) were diagnosed with cardiovascular disorder, 13.9 % (11 chronic obstructive pulmonary disease COPD, 13 bronchitis) were diagnosed with respiratory disorder, 25.4 % (31 diabetes, 10 hypothyroidism, 3 hyperthyroidism) were diagnosed with endocrine disorder, 4.6 % (5 agitatian, 3 delirium) neurological disorders, 6.9 % (6 metabolism, 6 acid reflux) had disorder of the GI, 3.5 % (6 renal failure) had urological disorder.

Most patients were treated for primary tumors (66.7 %), while the remaining patients were treated for recurrence (33.3 %). Approximately 56.2 % of the patients who were treated for primary OSCC had tumors in stage I or II, in detail were that: the tumor staging results were as follows: 148 in stage I, 262 in stage II, 113 in stage III, and 47 in unidentified.

According to the standards established by the American Joint Committee on Cancer (AJCC), the distribution results of the Tumor-Node-Metastasis criteria (TNM) [18] showed that 28.2 % of tumors were classified as T1 to T4. According to the gold standard for pathological analysis (H&E-stained tissue specimens and accompanying immunohistochemistry) [19]. And the final results were that: 148 in T1, 179 in T2, 47 in T3, and 139 in T4.

The main regions of HNSCC were as follows: 20 were in the lips (15 in lower lip, 5 in upper lip), 17 were in the maxillofacial region, 33 were in the floor of mouth, 122 were in tongue, 12 were in oral-pharyngeal, 12 were in hypopharynx, 49 were in neck, 52 were in the maxilla while 123 were in mandible, 65 were in buccal mucosa, the rest 6 were in the parotid gland. The classifications of the surgical operations were as follows: 149 in IO, 261 in JO, and 103 in SMO. Among the patients, 292 experienced postoperative complications.

The distribution of the postoperative complications (*n* = 292) were as follows: 58 had infections (35 wound infections, 23 free flap infection), 46 had edema (all in surgical region), 40 experienced wound dehiscence, 25 had hematoma (15 hematoma with re-exploration, 10 hematoma without re-exploration), 21 had flap necrosis (17 partial flap necrosis, 4 total flap necrosis), 20 respiratory disorder (19 pneumonia, 1 pulmonary embolus), 16 had a salivary fistula, 9 had gastrointestinal disorders (5 abdominal discomfort, 4 hematemesis), 8 had a central nervous system co-morbidity, 7 had a deep venous thrombosis (DVT) of the lower extremities, 6 had angina, and 8 had delirium. Detailed clinicopathological parameters of the external test set were summarized in Table 3.

**Table 3:** Clinicopathological characteristic of the patients in external test set

Characteristic	No.(%) of patients
Gender	Total: 12
Male	8(66.7 %)
Female	4(33.3 %)
Primary/recurrence	Total: 12
Primary	10(83.3 %)
Recurrence	2(16.7 %)
Co-morbidities	Total: 12
High blood pressure	3(25.0 %)
Myocardial infarction	5(41.7 %)
Diabetes	4(33.3 %)
Pathological grading	Total: 12
I	6(50.0 %)
II	4(33.3 %)
III	2(16.7 %)
Unidentified	0(0 %)
Clinical grading	Total: 12
T1	6(50.5 %)
T2	5(41.7 %)
T3	1(8.3 %)
T4	0(0 %)
Region	Total: 12
Floor of mouth	4(33.3 %)
Tongue	3(25.0 %)
Oral-pharyngeal	1(8.3 %)
Neck	1(8.3 %)
Mandible	1(8.3 %)
Parotid gland	2(16.7 %)
Surgical classification	Total: 12
IO	6(50.0 %)
JO	2(16.7 %)
SMO	4(33.3 %)
Postoperative complications	Total: 6
Wound infection	3(25.0 %)
Partial flap necrosis	1(8.3 %)
Total flap necrosis	1(8.3 %)
Pulmonary embolus	1(8.3 %)

### Variable were further selected according to POSSUM system, APACHE II system and clinical experience

According to the POSSUM system, 18 variables were selected: age, cardiac function, respiratory function, ECG results, systolic blood pressure (BP), pulse rate, hemoglobin levels, white blood cell (WBC) count, levels of blood urea nitrogen, sodium and potassium, Glasgow coma scale (GCS), operation type, number of procedures, operative blood loss, peritoneal contamination, malignancy, and Confidential Enquiry into Perioperative Deaths (CEPOD). With regards to the APACHE II system, nine variables were considered: rectal temperature, mean arterial pressure, blood pH, heart rate, respiratory function, levels of sodium, potassium and creatinine, hematocrit.

The following 17 “new variables” were chosen through clinical experiences and were evaluated by an SPSS17.0 toolkit: preoperative WBC, preoperative blood urea, diameter of the tumor, the amount of blood loss, operation time, hematocrit of postoperative day 1 (POD1), glucose of POD1, the chronic diseases, smoking, alcoholism, preoperative radiotherapy, preoperative chemotherapy, preoperative surgery, primary/recurrence of tumor, surgery classification, clinical grading, and pathological grading.

The 17 new variables were classified into the following 4 parameters: the preoperative variables, the operative variables, the postoperative variables, and the other variables. All of the above variables were classified into 4 tables: the preoperative variables are listed in Table 4, the operative variables are listed in Table 5, the postoperative variables are listed in Table 6, and the other variables are listed in Table 7.

**Table 4:** The preoperative variables are listed and evaluated by *P* and *F* values through SPSS 17.0 toolkit

Variable	Postoperative complication group	Non-postoperative complication group	*T*-value	*P*-value
Preoperative heart rate (beats/min)	75.25 ± 10.42	76.55 ± 12.13	1.298	0.132
Preoperative systolic pressure (mmHg)	149.14 ± 22.62	146.38 ± 23.36	1.358	0.843
Preoperative white blood cell count (×10^9^/L)	6.04 ± 1.86	6.46 ± 2.14	2.289	**0.043**
Preoperative hemoglobin (g/L)	127.00 ± 15.44	128.03 ± 16.93	0.713	0.712
Preoperative serum sodium (mmol/L)	143.17 ± 8.106	135.20 ± 6.00	1.136	0.095
Preoperative blood potassium (mmol/L)	3.30 ± 0.57	3.34 ± 0.62	0.628	0.410
Preoperative blood sugar (mmol/L)	5.13 ± 1.01	5.23 ± 1.10	0.942	0.957
Preoperative blood urea (g/L)	5.01 ± 1.53	5.52 ± 8.78	0.888	**0.044**

The two variables that were finally selected are highlighted

**Table 5:** The operative variables were evaluated by P and F values through an SPSS 17.0 toolkit

Group	Postoperative complication group	Non-postoperative complication group	*T*-value	*P*-value
Diameter of tumor (cm)	2.65 ± 1.54	3.49 ± 1.86	5.526	**<0.001**
The amount of blood loss (ml)	239.73 ± 231.40	606.29 ± 356.31	13.084	**0.001**
Operation time (hour, H)	3.19 ± 2.06	5.74 ± 2.69	11.996	**0.001**

The three variables that were finally selected are highlighted

**Table 6:** The postoperative variables were evaluated by P and F values through SPSS 17.0 toolkit

Variable	Postoperative complication group	Non-postoperative complication group	*T*-value	*P*-value
Temperature on the first day after operation (°C)	36.69 ± 0.47	36.89 ± 0.57	4.376	0.062
Heart rate on the first day after operation (beats/min)	80.19 ± 10.92	82.32 ± 10.89	2.208	0.381
Breathing rate on the first day after operation (breaths/min)	19.38 ± 2.38	19.20 ± 2.33	0.868	0.412
White blood cell count on the first day after operation (×10^9^/L)	11.89 ± 4.32	13.07 ± 4.09	2.916	0.075
Serum sodium on the first day after operation (mmol/L)	135.58 ± 4.59	135.56 ± 4.70	1.136	0.095
Blood potassium on the first day after operation (mmol/L)	3.36 ± 0.69	3.37 ± 0.58	0.011	0.844
Hematocrits on the first day after operation (l/L)	0.35 ± 0.04	0.33 ± 0.04	5.303	**0.045**
Serum creatinine on the first day after operation (μmol/L)	72.53 ± 20.32	74.97 ± 23.55	0.986	0.064
Blood sugar on the first day after operation (mmol/L)	6.83 ± 1.01	8.23 ± 1.10	0.982	**0.042**

The two variables that were finally selected are highlighted

**Table 7:** The other variables are listed and were evaluated by chi-square test and by Fisher's exact test with the SPSS17.0 toolkit

Variable	Postoperative complication group	Non-postoperative complication group	*P*-value
Primary			
Yes	169	165	**0.042**
No	84	95	
Chronic disease			
Yes	29	150	**<0.001**
No	224	110	
Smoking			
Yes	47	64	**0.049**
No	206	196	
Alcoholism			
Yes	32	35	**0.044**
No	221	225	
Preoperative radiotherapy			
Yes	14	31	**0.008**
No	239	229	
Preoperative surgery			
Yes	75	84	**0.028**
No	178	176	
Preoperative chemotherapy			
Yes	14	30	**0.011**
No	239	230	
Surgery classification			
IO	119	30	**<0.001**
MO	125	136	
JO	9	94	
Clinical Stage (TNM)			
T1	84	64	**<0.001**
T2	84	95	
T3	23	24	
Pathology Stage			
I	84	64	**0.045**
II	125	137	
III	23	24	

The 10 variables that were finally selected are highlighted

With the above variables, 4 types of variable systems were formed: the “POSSUM Variable” system, the “APACHE II Variable” system, the “NEW Variable” system, and the “All (All = POSSUM + APACHE + NEW) Variable” system.

### The accuracy and reliability of each predictive model were evaluated under 4 different “All Variable” systems

**Fig. 1:**
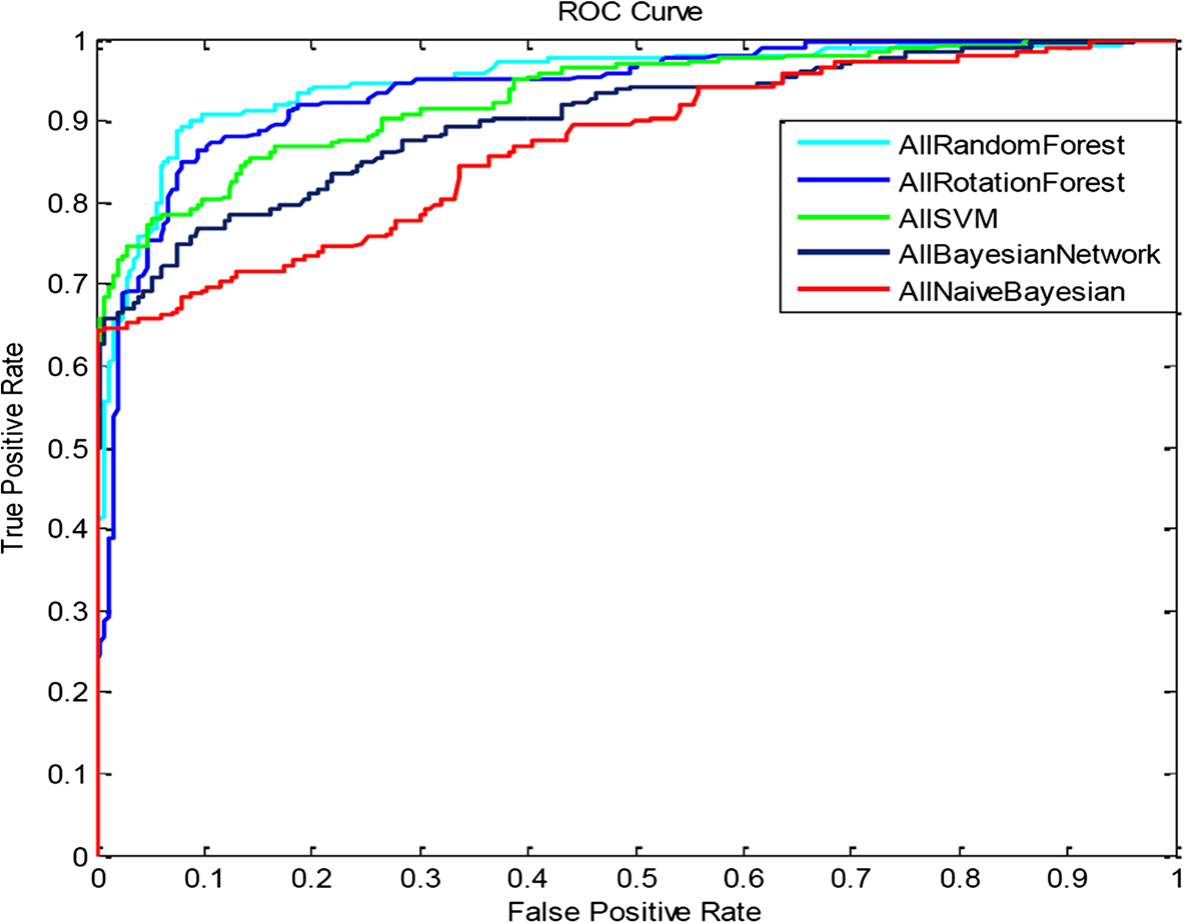
There are 5 ROC curves that illustrate the reliability of the predictive models based on 5 data mining algorithms under the “All Variable” system. The light blue curve signifies the predictive model based on the Random Forest algorithm, which has the largest AUC value. All AUC values are shown in Table 9. SVM = Support Vector Machine

In this study, five different data mining algorithms were used to construct predictive models. Eventually, five predictive models based optimal parameters selection were successfully constructed. Also it has been validated that the accuracy of each predictive model under four different variable systems. The performance of each predictive model is listed in Table 8. According to Table 8, each predictive model had the best accuracy under the “All variable” system. In addition, the predictive model that was based on the Random Forest algorithm had the best accuracy (89.084 %). Since the comparison among accuracy of each predictive model under the “All Variable” system was not comprehensively enough, it was necessary to know the reliability of each model under the “All Variable” system. The ROC curve was adapted to detect the difference in the reliability of the sensitivity of each predictive model under the “All Variable” system. The ROC of each predictive model under the “All Variable” system is shown in Fig. 1. The difference between each area under the curve (AUC) is shown in Table 9, which suggests that the predictive model based on the Random Forest algorithm generated the best AUC value of 0.949, which in turn, indicates the greatest reliability. Accordingly, It is verified that the difference performance among these data mining algorithms may come from the following reasons: random forest algorithm is one of the strongest algorithm in the classification problems, and moreover, these clinical data naturally have the distribution suitable for decision tree modeling, which is the basis model of random forest algorithm.

**Table 8:** To find the accuracy of each predictive model under different variable systems, a 5-fold-cross validation was used

Algorithm variable	Accuracy				
	Support Vector Machine (SVM)	Random Forest (RF)	Rotation Forest (ROF)	Bayesian Network (BN)	Naïve Bayesian Network (NBN)
All^a^	83.431 %	89.084 %	85.965 %	82.261 %	75.634 %
New^b^	81.676 %	87.135 %	83.041 %	77.778 %	73.489 %
POSSUM^b^	79.337 %	82.261 %	79.142 %	74.074 %	71.929 %
APACHE II^b^	75.439 %	76.420 %	76.023 %	73.294 %	75.829 %

The predictive model based on the Random Forest algorithm has the best accuracy of 89.084 % under the “All variables” system

^a^All variables included in the study

^b^Variables included in the model

**Table 9:** The AUC of each predictive model under “All Variable” system, and the predictive model based on random forest algorithm has the largest AUC value of 0.949

Variable	AUC
All Random Forest	0.949
All Rotation Forest	0.942
All SVM	0.930
All Bayesian Network	0.905
All Naïve Bayesian	0.865

*All* ”All Variable” system

*SVM* “Support Vector Machine” algorithm

### The accuracy and reliability of the Random Forest algorithm model were among the best and tested in an external testing set

**Fig. 2:**
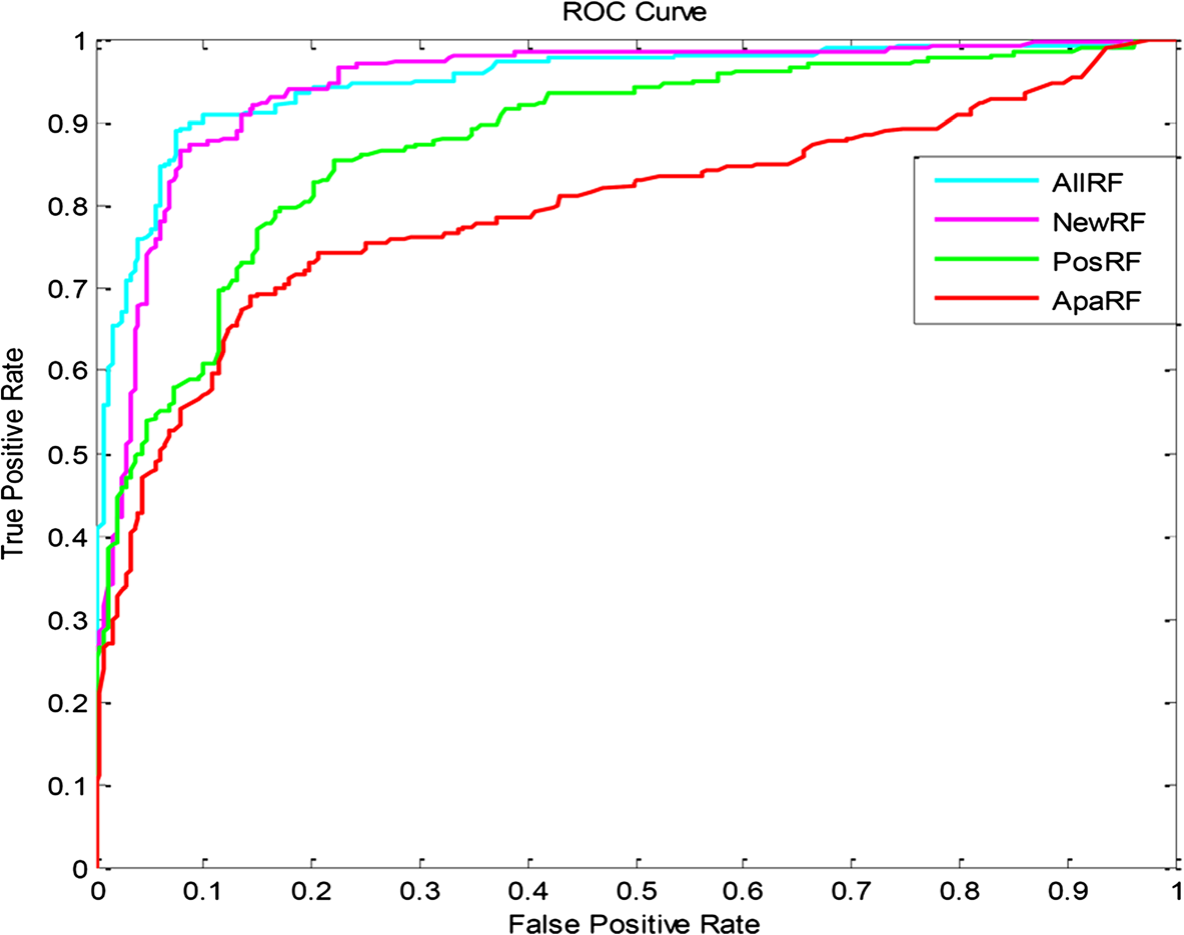
There are 4 ROC curves that illustrate the reliability of the predictive models based on the Random Forest algorithm under 4 different variable systems. The light blue curve signifies the predictive model based on the Random Forest algorithm under the “All Variable” system, which has the largest AUC value. All AUC values are shown in Table 10. *All*=”All Variable” system, *New*=”New Variable” system, *Pos*=”POSSUM Variable” system, *Apa*=”APACHE II Variable” system, *RF* = Random Forest algorithm

Our findings show that the predictive model based on the Random Forest algorithm under the “All Variable” system generated the best accuracy as well as the best reliability in the training set (*n* = 513). Furthermore, the performance of the Random Forest algorithm model was calculated under all 4 different variable systems. In Fig. 2, the ROC of the predictive model under the 4 different variable systems is shown. And the AUC values for Fig. 2 are listed in Table 10, which shows that the predictive model based on the Random Forest algorithm under the “All Variable” system, had the largest AUC value (0.949). This means that the Random Forest algorithm model under the “All Variable” system still produce the best reliability. In order to further test the accuracy and reliability of the Random Forest algorithm model under the “All Variable” system, an external test set (*n* = 12) was incorporated into the study, our results demonstrated that the model gave a Performance (accuracy = 83.333 % and the AUC value = 0.781), which was also showed in Table 11.

**Table 10:** The AUC of the predictive models based on the random forest algorithm under 4 different variable systems, and the predictive model under the “All Variable” system has the largest AUC value of 0.949

Variable	AUC
All RF	0.949
New RF	0.944
POSSUM RF	0.878
APACHE II RF	0.794

*All* “All Variable” system

*New* “New Variable” system

*POSSUN* “POSSUM Variable” system

*APACHE II* “APACHE II Variable” system

*RF* random forest algorithm

**Table 11:** Comparison between the training variable set and the external variable set

Variable set (Training)	CA (Accuracy, %)	AUC
All Random Forest	89.084 %	0.949
All Rotation Forest	85.965 %	0.942
All SVM	83.431 %	0.930
All Bayesian Network	82.261 %	0.905
All Naïve Bayesian Network	75.634 %	0.865
Variable set (external)		
All Random Forest	83.333 %	0.781

*All* “All Variable” system

*SVM* support vector machine

Even if there are rarely algorithm analysis references for the study of postoperative complications with HNSCC, the obtained prediction result (83.333 %) is also a promising result compared to algorithm results from similar clinical data modeling, which bridge the gap between clinical data and advanced algorithm modeling.

## Discussion

The increase in life expectancy has resulted in an increasing number of malignant neoplasms in the elderly population [20]. Most head and neck squamous cell carcinoma (HNSCC) commonly arises between the fifth and seventh decades of life; therefore, their occurrence in the elderly population is not uncommon [20]. Surgery and/or radiotherapy are the primary locoregional treatment modalities [21] and are often followed by chemotherapy [22]. The postoperative complications are among the most difficult factors in the surgical management of elderly patients with HNSCC. HNSCC in elderly patients should be treated with curative intention [23]. In this study, the authors emphasize the importance of the prediction of postoperative complications.

Postoperative complications that occur within the first 24 h during or following surgery are regarded as early, and those that occur within the 2-week postoperative period are considered delayed [24]. Some postoperative complications with a high rate of occurrence that may elicit serious effects are as follows: 1) pulmonary embolus, diagnosed by a high-probability nuclear ventilation-perfusion scan or by pulmonary angiography, is a common complication that may also cause venous thromboembolism [24], and 2) a morbidity within the central nervous system, which is defined as a newly documented cerebrovascular accident, transient or reversible neurologic deficit, or neurologic deficits of any origin, including those related to ethanol withdrawal [24]. These may lead to the failure of behavior management, which has been a negative factor for the treatment of free flaps, possibly causing flap necrosis. 3) Infections of the wound and flap caused by the invasion of bacteria or foreign bodies to the wound or flap may be detected after a blood analysis; this is especially true after a WBC count, which can help determine the causes of fever and of the necrosis of the free flap or skin graft. 4) Deep venous thrombosis of the lower extremities, which is recognized as a cause of both pulmonary embolism (PE) and of post thrombotic syndrome [25], may cause sudden death in this patient population. Edema, wound dehiscence, hematoma under the skin, flap necrosis, salivary fistula, gastrointestinal disorders, and angina pectoris are also among the most common postoperative complications.

As a result, 5 different data mining algorithms were exploited to construct predictive models. It had also been validated that the accuracy of predictive model under four different variable systems. The predictive models based on the random forest, the rotation forest, and the support vector machine algorithms are the most accurate under the “All Variable” system (89.084, 85.965, and 83.431 %, respectively). Additionally, the random forest algorithm was regarded as the most suitable algorithm model in this study. The reliability of each model under the “All Variable” system was obtained through the ROC curve, then each area under curve (AUC) was also calculated; Subsequently the predictive model based on the Random Forest algorithm had the largest AUC value of 0.949, which suggested the best reliability. Furthermore, the performance of the random forest algorithm model was verified in an external testing set (accuracy = 83.333 % and the AUC value = 0.781).

Based on the results, there are some weaknesses and challenges in the study. First, our study is limited by its reliance on data from a single hospital, and thus the number of patients is small. Second, the study lacks input variables for patient variables, such as exercise behavior, nutrition, stress, and depression. Third, the quality may be impacted because the number of non-postoperative complication group was higher than the postoperative complication group. Finally, for data mining, the quantity and quality of the data are so important. Therefore, a multi-center study including more variables for prediction of postoperative complications by using the random forest algorithm model warrants further investigation.

In this study, for the first time, we demonstrated that the potential of computational prediction model of postoperative complications in elderly patients with HNSCC was founded by using data mining approach. The random forest algorithm model generated the best accuracy (89.084 %) and the best reliability (AUC = 0.781) in a training set, which was further verified in an external testing set (83.333 % and AUC = 0.781), suggesting the random forest algorithm would be a promising prediction model for postoperative complications in elderly patients with HNSCC.

## Conclusions

Data mining (DM) techniques allow the possibility of statistically predicting postoperative complications in elderly patients with HNSCC. In the study, the Random Forest algorithm model provides a promising approach with the best accuracy (89.084 %) and the best AUC value (0.949). This was further verified in an external testing set, with a good accuracy (83.333 %) and the AUC value (0.781).
